# Implementation of repeat HIV testing during pregnancy in Kenya: a qualitative study

**DOI:** 10.1186/s12884-016-0936-6

**Published:** 2016-07-11

**Authors:** Anna Joy Rogers, Elly Weke, Zachary Kwena, Elizabeth A. Bukusi, Patrick Oyaro, Craig R. Cohen, Janet M. Turan

**Affiliations:** Department of Health Care Organization and Policy, University of Alabama at Birmingham School of Public Health, Birmingham, USA; Centre for Microbiology Research, Kenya Medical Research Institute, Nairobi, Kenya; Department of Obstetrics, Gynecology & Reproductive Sciences, University of California, San Francisco California, USA

**Keywords:** PMTCT, Pregnancy, HIV counseling and testing, Kenya, Guideline implementation

## Abstract

**Background:**

Repeat HIV testing in late pregnancy has the potential to decrease rates of mother-to-child transmission of HIV by identifying mothers who seroconvert after having tested negative for HIV in early pregnancy. Despite being national policy in Kenya, the available data suggest that implementation rates are low.

**Methods:**

We conducted 20 in-depth semi-structured interviews with healthcare providers and managers to explore barriers and enablers to implementation of repeat HIV testing guidelines for pregnant women. Participants were from the Nyanza region of Kenya and were purposively selected to provide variation in socio-demographics and job characteristics. Interview transcripts were coded and analyzed in Dedoose software using a thematic analysis approach. Four themes were identified a priori using Ferlie and Shortell’s Framework for Change and additional themes were allowed to emerge from the data.

**Results:**

Participants identified barriers and enablers at the client, provider, facility, and health system levels. Key barriers at the client level from the perspective of providers included late initial presentation to antenatal care and low proportions of women completing the recommended four antenatal visits. Barriers to offering repeat HIV testing for providers included heavy workloads, time limitations, and failing to remember to check for retest eligibility. At the facility level, inconsistent volume of clients and lack of space required for confidential HIV retesting were cited as barriers. Finally, at the health system level, there were challenges relating to the HIV test kit supply chain and the design of nationally standardized antenatal patient registers. Enablers to improving the implementation of repeat HIV testing included client dissemination of the benefits of antenatal care through word-of-mouth, provider cooperation and task shifting, and it was suggested that use of an electronic health record system could provide automatic reminders for retest eligibility.

**Conclusions:**

This study highlights some important barriers to improving HIV retesting rates among pregnant women who attend antenatal clinics in the Nyanza region of Kenya at the client, provider, facility, and health system levels. To successfully implement Kenya’s national repeat HIV testing guidelines during pregnancy, it is essential that these barriers be addressed and enablers capitalized on through a multi-faceted intervention program.

## Background

The integration of HIV testing into antenatal care settings has been a key contributor to the decline in mother-to-child transmission (MTCT) of HIV. Of the 22 priority countries identified by the Joint United National Programme on HIV/AIDS (UNAIDS) that account for over 90 % of all MTCT, as of 2012 seven had achieved testing rates of over 90 % of pregnant women and 14 had achieved at least 50 % [1]. While this is encouraging, a recent meta-analysis found the pooled cumulative incidence of new HIV infections during pregnancy and the postpartum period in African countries is 3.6 % (95 % CI: 1.9–5.3 %), suggesting that a single antenatal test may fail to capture an important subset of women who acquire HIV during this period and whose infants are at high risk of HIV acquisition due to elevated viral loads associated with acute HIV infections [2]. Additionally, as women with prevalent infection are increasingly identified at the first antenatal visit, it is estimated that 34 % of all MTCT in the future will be among women with incident infection after the first antenatal care (ANC) clinic visit [3].

Experts have called for HIV re-testing in late pregnancy, a recommendation that has been adopted by the international elimination of MTCT agenda [2, 4]. In Kenya, repeat HIV testing in the ANC setting, defined as retesting three months after initial presentation at antenatal clinic, is national policy [5]. Although more than 90 % of pregnant women in Kenya receive an initial HIV test and research suggests that retesting acceptability is high [6], current rates of retesting among pregnant women in Kenya are unknown. There is a lack of data on the implementation of repeat testing in sub-Saharan Africa; only one known observational study, conducted in Zambia, reports the rate of repeat HIV testing during pregnancy to be 24.5 % at a district hospital [7].

Much research has been done on the barriers to initial HIV testing in sub-Saharan Africa among the general population, identifying factors such as stigma, lack of information, perceptions of lack of privacy and confidentiality, poor relationships with health staff, and fear of being HIV-positive [8, 9]. However, the barriers and enablers to repeat HIV testing among pregnant women who have already accepted HIV testing once are less clear [10].

In order to address these gaps in the literature, we carried out 20 qualitative semi-structured in-depth interviews with administrators and providers of healthcare to explore the barriers and enablers to retesting pregnant women for HIV in rural Kenyan health facilities. It is anticipated that this research will help inform the design of a multi-faceted intervention to improve implementation of the HIV repeat testing policy for pregnant women in Kenya and other similar settings globally.

## Methods

### Setting and context

Data were gathered in the Nyanza region of Kenya, which has the highest prevalence of HIV in the country at 15.1 % [11]. Although much of the population lives in rural areas, 96 % of pregnant women attended at least one antenatal care appointment in 2012 and 93.1 % received at least one HIV test, making antenatal care clinics an important site for HIV testing and linkage to HIV treatment [12]. Of the three study sites, all of which were located in rural areas, one site was in a mining community, another in a farming community, and the last in a community that engages in a range of income-generating activities.

### Study design

Ethics approval was given by the Kenya Medical Research Institute Ethical Review Committee and the University of Alabama at Birmingham Institutional Review Board. A qualitative in-depth interview guide was developed based on a review of the literature assessing the common barriers and enablers to HIV testing in all populations. Participants were identified from three ANC clinics affiliated with Family AIDS Care Education and Services (FACES) [13], a CDC-PEPFAR funded initiative that supports government health facilities in providing comprehensive HIV prevention, care, and treatment services. Twenty healthcare providers and managers were chosen from a sampling frame of all potential participants at the study-approved sites, purposively selecting them for variation in socio-demographics and job characteristics. A sample size of 20 was chosen in order to include the perspectives of different types of providers and managers working at the study sites. Data saturation was achieved in 20 interviews, indicating that the sample size was sufficient for this qualitative study. Types of participants interviewed included nurses, community health workers, health educators, HIV testing counselors, laboratory technicians, facility coordinators, FACES program technical advisors, trained lay healthcare workers, and administrative staff involved in finances and procurement.

Participant demographic and job characteristics were collected using a standard questionnaire. A single interviewer (AJR) conducted in-depth interviews using the semi-structured interview guide, which had been pilot tested for question clarity with two volunteers. All interviews lasted approximately one hour and were conducted in English, a national language of Kenya in which most healthcare providers are fluent. Following signed informed consent, participants were interviewed in a private setting and reimbursed 400 Kenyan Shillings (roughly equivalent to US $5) as compensation for their time. Interviews were digitally recorded and transcribed verbatim by experienced transcriptionists without identifying information.

### Data coding and analysis

Interview transcripts were coded and analyzed by a single researcher (AJR) using the Dedoose qualitative software program (SocioCultural Research Consultants, LLC; Los Angeles, California). The coding and analysis were conducted using a thematic analysis approach [14]. Four initial major themes were identified using Ferlie and Shortell’s Framework for Change, which posits that change can be focused at the individual level, the group or team level, the organizational level, and the larger system or environmental level, and adapted to the Kenyan setting [15, 16]. Additional sub-themes were allowed to emerge from the data, and categorized into being either ‘enablers’ or ‘barriers’ to retesting.

## Results

### Participant characteristics

All 20 of the healthcare providers and managers approached agreed to participate. Sixty-five percent self-identified as healthcare providers with the majority of their time spent engaging with patients; the remainder primarily fulfilled managerial or administrative roles. The average age was 34 years, the average time in current job was 4.7 years, and 35 % were female. In terms of the highest level of education, 15 % (3 participants) had completed high school or less, 45 % had a certificate or diploma, 25 % had a bachelor’s degree, and 15 % had a master’s degree or higher (Table 1).

**Table 1:** Interview participant characteristics

Participant characteristics (*n* = 20)	Proportion or Average
Job Type	
Healthcare provider	65 %
Managerial or administrative	35 %
Gender – Female	35 %
Age (average, in years)	34
Time in Current Job (average, in years)	4.7
Highest Level of Education	
Form 4 Completion or less	15 %
Certificate or Diploma	45 %
Bachelor’s Degree	25 %
Master’s Degree or higher	15 %

### Barriers

**Fig. 1:**
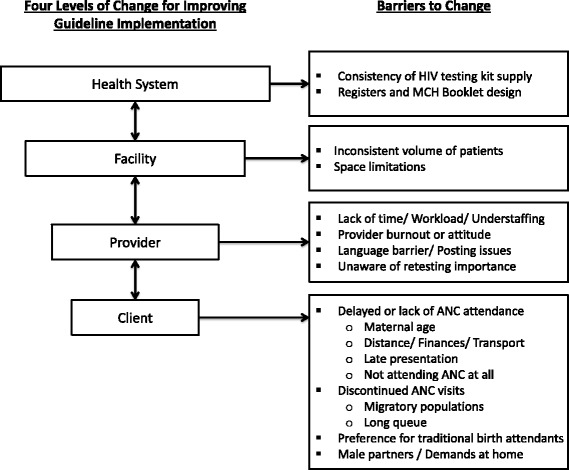
Barriers to Improving Guideline Implementation at Four Levels of Change. Figure legend: Adapted from Shortell [15] and Proctor et al. [37]. Used with permission

Participants identified barriers to improving guideline implementation at four levels of change: the client, provider, facility, and health system levels (Fig. 1).

### Barriers at the client level

Providers generally acknowledged that repeat HIV testing rates were low among women attending their clinics, with their estimations of the proportion getting tested at their clinic varying from 30 to 100 %. Client-level barriers to a higher retesting rate discussed by the providers fell into four main categories: (A) factors influencing a late initial presentation for ANC or the decision to not come to clinic at all (B) factors influencing discontinued antenatal clinic visits after their initial visit, (C) preference for community-based services from traditional birth attendants, and (D) male partner factors.

#### Factors influencing delayed initial presentation or antenatal clinic non-attendance

##### Maternal age

While pregnant women of all ages attended the clinics, some providers sensed that different issues might hinder younger and older women from attending clinic. According to one nurse, lack of knowledge and anticipated stigma resulted in late initial ANC presentation, especially for young women.

“Especially the young girls, they tend to really hide… and most of them are actually forced to come to the clinic… They are reluctant to come because [for] one thing they are not aware they are supposed to start antenatal clinic; they are young mothers so they don’t even know. They are pregnant but their main worry is not how the baby will be [but] it is just what people will say, … and then maybe they fear that probably they will be expelled from school because that is what happens most of the time… probably they come as late as 8 months (#4, female, nurse).”

Conversely, older women may have different reasons for avoiding the clinic, including feeling like “if her daughter comes to the clinic here and then she also comes to the clinic… it wouldn’t be a good picture [because] she is still giving birth and the daughter is also giving birth (#1, female, trained lay healthcare worker).” Alternatively, older women of higher parity may feel “knowledgeable enough (#14, female, health educator)” to skip antenatal appointments.

##### Distance/Financial strain/Lack of transportation

Distance to the clinic – and by extension the lack of transportation or finances to pay for transportation – influenced when women began antenatal clinic or whether they came for more than one appointment. Some participants felt that financial barriers were a definite hindrance; one provider commented that some women could barely afford to pay for food, much less transportation. In addition, time spent at the clinic is lost income. Said a community health worker, “They feel that ‘If I went to dig for somebody the garden I would get 200 shillings but if I go to the clinic I will not get a single cent.’ (#11, male, community health worker).” Other participants disagreed that financial barriers could hinder attendance, particularly with the introduction of free antenatal and delivery services at government health facilities, and because women had come to value their appointments.

##### Late presentation

One of the most raised challenges to antenatal care in general, and to the recommended frequency of HIV testing in pregnancy in particular, was the late stage of pregnancy at which many women present for their first visit, which is commonly after the first trimester. If tested after 28 weeks, they will not be eligible for a retest during antenatal care or delivery and may be subsequently lost to follow up in the postpartum period. Participants posited that several factors may contribute to this phenomenon. Some women may not realize that they are pregnant. They may not track their menstrual periods and may fail to notice or associate a missed period with pregnancy, particularly if they are without access to a pregnancy test. There may also be cultural issues associated with openly acknowledging pregnancy:

“Pregnancy is a private thing. There are people who when they are pregnant, they are stigmatized… They even don’t share with the husband for quite some time, even after the pregnancy is four, five months… There are people who would wait for the pregnancy to be visible before they come. That is when now they believe that they are really pregnant (#3, male, community health worker).”

##### Not attending antenatal clinic at all

A subset of women may have unique reasons for avoiding clinic altogether. They include women who had an uneventful first pregnancy and assume that all subsequent pregnancies will be similarly smooth, women who do not consider pregnancy to be a “sickness” requiring a trip to a hospital, and women who belong to religions forbidding visits to health facilities. One provider commented, “There are people because of their religion they are not allowed to go to the health facility. Like we have the ‘Legio Mariae,’ a church that with their belief people [in this area of Kenya] don’t go to the facility (#1, female, trained lay healthcare worker).”

#### Factors influencing discontinued ANC visits

##### Migratory populations

Participants in two of the facilities described that one major challenge to a higher retesting rate was the migratory nature of employment in the surrounding areas. One manager noted that this complicated antenatal care in general, and HIV repeat testing in particular, because “chances are you are seeing this woman today and you’ll never see her again (#18, male, nurse, FACES program technical advisor).” One provider noted the influx of women associated with new companies moving into the locale, while another reflected on the population in his service area:

“[I have seen] some new faces even of women… who are not married or are sex commercial workers, they go where there is money, they float around (#7, female, trained lay healthcare worker).”

“[Some women] come for mining purposes, stay there for almost two months or three, you know, the mining also has its season. There are seasons that it’s booming; [but a rainy] season like now, it’s now low. Some clients now migrate towards the lake for fishing and even some go home for farming … depending on how [they are] generating the income (#12, male, health educator).”

##### Long queue

As is the case in many clinics, patients are not given appointment times but rather come in the morning and wait to be seen. Women, particularly those with young children in tow, may struggle with the long wait associated with some clinic days. Several participants raised this issue and one health educator in particular commented:

“The clients… will wait until they become tired… due to lack of the staffs they may stay there for long before they are seen. So they will be waiting, their children get crying, so these mothers will start complaining… That may bring a challenge to those mothers because if you come to the clinic at eight, then you will leave the clinic even at twelve (#14, female, health educator).”

If women have had a frustrating waiting experience, this may negatively influence their desire to return. One participant expressed the sentiment that some women may experience:

“[When] they come, they find that there are already twenty people in the queue and you have to wait to be attended to so those are some of the things that make them really feel that they should not come to the clinic.” (#11, male, community health worker)

#### Preference for traditional birth attendants

Several participants explained that in many rural areas, traditional birth attendants (TBAs) are respected members of the community who provide labor and delivery services. In addition to potentially spending more time with laboring mothers than do clinic-based providers and using traditional medicine, which may be more in line with an expectant mother’s belief system, one manager summarized the myriad of other reasons succinctly:

“Personalized care, comfort, the home environment, rumors about the hospital… The traditional birth attendant gives extra things like… tea and porridge once they deliver. The traditional birth attendants can be paid in kind. They can be given chicken… instead of money. She’s someone you know from the village and you trust her and you want to have your baby with someone you trust, someone you know. (#18, male, nurse, FACES program technical advisor).”

TBAs seldom provide antenatal care services, so women may initially attend clinic and get tested for HIV, but ultimately decide to have a home-based birth with a TBA. Labor is an important time to emergently intervene on HIV transmission, and given that 28 % of women rely on TBAs for delivery services [17], the lack of repeat HIV retesting services by many TBAs may limit their ability to prevent mother-to-child transmission.

#### Male partners/Demands at home

Expectant mothers may have limitations placed on them by their male partners or their responsibilities at home. One provider commented that male partners may even forbid their wives from attending clinic. Other providers commented that women may be afraid to test for HIV without their partner’s consent or presence. On the other hand, some male partners are supportive of women attending clinic, partially because they know that HIV testing is routine during antenatal care. At least three participants agreed with a provider who said,

“I know the male partner; they will be happy once they get to know [the pregnant woman’s HIV] status. …They take it as once the partner is negative they already know their status that they are negative (#2, male, laboratory technician).”

One provider even commented that men “sneak look at the mother-baby booklet [a maternal and child health record], see the [HIV] result and interpret that to be their own result (#4, female, nurse).” Pregnant women also have competing demands at home. They may fear that if they spend half a day traveling to, waiting at, and returning from the clinic, they will neglect their tasks and raise the ire of their husbands.

### Barriers at the provider level

#### Lack of time/High workload coupled with understaffing

The most frequently cited provider-level hindrance to retesting for HIV was the problem of a high patient volume, coupled with insufficient time to dedicate to each patient. Two main coping mechanisms were discussed – a nurse described postponing testing and an HIV testing counselor discussed pressure to cut counseling sessions short:

“At times it is so hectic. You are one person and you have to test clients, probably they are several [waiting to be] tested at the initial test and others who come for retest. So probably you will just postpone the one for the retest because you have too much work load (#4, female, nurse).”

“You need to have time with somebody and provide an environment where somebody can freely speak of the true issues that are challenging to him or her… [but] there is a queue out there with angry clients who are feeling that you are taking a lot of time (#5, female, HIV testing counselor).”

Thus, several providers commented that a high workload may be associated with a decrease in the quality of HIV-testing services that are offered. However, participants expressed that HIV retesting does take less time than the initial test since the counseling portion is less comprehensive. Some providers felt like ANC clinics were understaffed and that staff was expected to provide too many services (ANC, delivery services, postnatal care, and child welfare clinic), particularly in smaller facilities.

#### Burnout/ Attitude

Related to the issue of workload and pressure is the problem of provider burnout. As one participant described, counseling is different for each client and can be mentally draining:

“There is burnout. The staff who is offering the service is already tired probably she has done HIV counseling and testing for 30 people. They’ve talked, they are very tired. You can imagine if one tests HIV negative it’s different from when one tests positive because when one tests HIV positive there is that psychosocial support that comes with the counseling and therefore the counseling is prolonged (#3, male, community health worker).”

In spite of the integration of HIV services into regular clinic flow and the fact that most providers are trained in HIV testing and counseling, some providers still felt that HIV testing was the purview of dedicated HIV counselors. Leaving the responsibility of HIV testing to those individuals may result in women going untested when client volume is heavy.

#### Language barrier/Posting

One unanticipated barrier to providers offering HIV retesting services stems from the nature of job postings in rural areas. On occasion, these areas may not have local staff that are trained at the level of nurses or clinical officers, so these providers have to be hired from a different region of the country. This poses issues including language barriers, long commutes for providers who choose to stay in the nearest city center, and sometimes, “many are not ready to work in this region… they come and see the terrains and they just go back (#11, male, community health worker).” The impact of this challenge extends far beyond the issue of HIV repeat testing.

#### Unaware of retesting importance

While all the participants interviewed for this study demonstrated a clear understanding of the importance of retesting and stressed its necessity, they cautioned that not all providers may appreciate it in the same way. Some providers stated that they had personal experience with women seroconverting later in pregnancy, but that not all nurses or counselors would have had that experience. One participant described the thinking of some providers: “The retest may also be assumed as a waste of resources: ‘this woman has already tested, she knows her HIV status, why do we test again?’ (#11, male, community health worker)”

### Barriers at the facility level

#### Inconsistent volume of patients

Unlike in facilities or departments where patients are booked for appointments, clients show up at rural antenatal clinics on days that suit them best, especially if it is their initial visit. Participants frequently mentioned that some days – in particular market days on which clients are already traveling to town for selling or buying purposes – had a much heavier clinic volume. Additionally, most women tended to start lining up early in the morning, with few or no women coming in the afternoon. One provider said that this had to do with local beliefs:

“Something set in the mind in the community that for antenatal care you have to go in the morning… there is a myth in [the local language] Dholuo that when you go after eating ugali [the staple food] at noon… the nurse wouldn’t hear the baby but would hear the ugali in the stomach (#1, female, trained lay healthcare worker).”

#### Space limitations

Due to the confidential nature of HIV testing and counseling, a private space is essential. Participants commented on the fact that some clients decline testing if they have confidentiality concerns. Lack of space can also hamper a team-based approach to care, where overworked providers can call in back-up HIV testing counselors to concurrently attend to patients who are waiting. Makeshift rooms – such as tents or storage areas – are sometimes used in these facilities for HIV testing purposes. As one community health worker commented:

“Lack of space is a major, major, major issue. You’ll find a whole MCH [maternal and child client group] with a very congested room. This is the place where you do palpation [of the uterus for fundal height and] you want to do testing. It compromises confidentiality a lot. So space is an issue. Or even counseling session will be done in public or you do it in a group which is not very sufficient (#11, male, community health worker).”

### Barriers at the health system level

#### Consistency of HIV testing kit supply

Nearly all providers mentioned HIV testing kit shortages as a major challenge to consistently providing repeat HIV testing for pregnant women. As one community health worker commented:

“There were times when you can go around three weeks without the test kits. So it was a major challenge because the mothers will come back but with no test kits you cannot test them. You will again rebook them [for a new appointment]. There was a time when it went throughout the month without testing (#13, male, trained lay healthcare worker).”

When asked which clients would be prioritized in the event that it was necessary to ration remaining test kits, providers almost unanimously stated that pregnant women were a priority over patients in the outpatient or inpatient wards. As one manager commented, “We prioritize… the baby who is not born because we want to eliminate transmission of [HIV from] mother to child (#6, male, facility manager).” However, when faced with an expectant mother needing an initial test or a retest, providers reported that they would choose to forgo the retest.

Due to the complex system of reporting, approval, and distribution, delays in delivery of test kits can range from a few days to a month or more. Participants had difficulty pinpointing a single source of delay. One manager acknowledging potential fault on the facility side, but also mentioned that the number of kits ordered frequently do not match up with the number delivered: “One [reason for test kit shortages] is reporting. If the flow of reports is not good, back to the national system, then there could be delays. [Additionally] you order 100 and you’re given 50, it would be an issue (#17, male, FACES program manager).”

#### Registers and MCH booklet design

Since HIV testing is not a service that is offered at every antenatal care visit – unlike palpating the expectant mother’s abdomen to determine fundal height – determining eligibility for HIV retest is something that the provider must remember to initiate. However, this process is not easy given the nature of the medical records. These registers, designed by the National AIDS and STI Control Programme (NASCOP), record each patient visit sequentially in one book organized by visit date, rather than longitudinally for each individual patient. While this design may be optimal to allow for uniform service delivery and outcome reporting, it requires provider effort to flip back and forth through the register to find prior patient visit data. One FACES program technical advisor commented:

“From the register it’s difficult to answer the question ‘Who should be retested?’ because of the way the register is limited and its design… Maybe in the future if electronic registers can be designed in such a way that we can be able to determine eligibility for retesting then people would be sensitized and they know [it is time to retest]. And we can even give feedback and tell them this month we had 50 people eligible for retesting and we only tested 5; what could have gone wrong? But right now the way things are we can’t do that easily. It will take you a lot of time (#18, male, nurse, FACES program technical advisor).”

Additionally, while the Maternal and Child Health (MCH) booklet kept in possession of the woman is a useful clinical tool for longitudinally tracking the health of the woman and her child, it does not have dedicated space for multiple HIV test results.

### Enablers

#### Enablers at the client level

##### Motivations for attending antenatal clinic

Providers suggested that it was important to understand the motivations that their clients had for attending antenatal clinic, in order to encourage early and continued visits. The reasons they gave for why pregnant women may attend ANC included concern for the health of their infants, encouragement from other women in their peer group, successful facility marketing of free antenatal care and delivery services, or because they had benefited from attending antenatal care for previous pregnancies. Others felt like being able to receive preventative testing and medications (such as for syphilis or malaria); or gifts like t-shirts, insecticide-treated nets, and lessos (traditional cloth wraps) were the main incentives.

##### Beliefs about PMTCT possibility

Participants also emphasized many expectant mothers are beginning to see the fruits of successful prevention of mother-to-child transmission (PMTCT) efforts among their HIV-positive acquaintances. One manager shared the power of positive testimony:

“They used to know that once you are HIV-positive automatically the child will come out HIV-positive… they were seeing it as something that is obvious [and] expected. But of late once they have started to hear and have seen others who have gone through PMTCT [who] have come out with babies who are HIV-negative. Now the mothers [who are] HIV-positive want to know how she is going to get an HIV-negative child (#9, male, facility manager).”

Thus, word of mouth was described as a powerful enabler of HIV retesting programs.

#### Enablers at the provider and facility levels

Participants felt that that cooperation and task redesign may help implement change at the provider team and facility levels. They recommended that all healthcare providers working in maternal and child health should be trained on HIV testing and counseling and work together as a team when the patient volume is heavy. For example, one provider said, “…Let them all be trained on testing so that we don’t miss an opportunity because one of the healthcare providers doesn’t know how to test (#5, female, HIV testing counselor).” A change in the organizational culture may be required, such that providers no longer see some duties as “a responsibility of such and such a person (#11, male, community health worker).” Additionally, one participant commented that strategically placing motivated staff was important: “We’ve identified dedicated staffs to be champions… so other staffs see the way they work and now they feel motivated and say ‘Eh kumbe hata sisi we’ – [meaning] even us we are able to do it (#20, male, administrator).”

#### Enablers at the health system level

Numerous enablers to retesting were identified at the health system level, particularly pertaining to a steady supply of HIV test kits. One administrator involved in procurement (#19) commented that timely funding disbursement, tight collaboration between donor agencies and various branches of the government, and accurately projected budgets were crucial for consistent supplies. Another administrator involved in finances and procurement (#16) applauded the move from a paper-based to electronic format of ordering supplies, as this not only improved speed of orders reaching supply warehouses, but also accuracy of reporting.

## Discussion

Previous literature has documented the barriers to an initial antenatal HIV test among pregnant women, as well as barriers to HIV retesting in other populations who have been tested at least once [8–10]. This study focused on the barriers to repeat HIV testing among pregnant women in sub-Saharan Africa, a strategy that experts have called for to address incident HIV during pregnancy and the associated high risks of HIV-related maternal mortality and MTCT to infants [2]. The main finding of our study is that implementing a higher retesting rate will require a multi-faceted approach and a successful strategy will likely address barriers that exist on four levels, as represented in Ferlie and Shortell’s Framework for Change: the client, provider, facility, and health system levels. The issue of low repeat antenatal testing rates is not isolated from other clinic performance indicators. Therefore, if some of the barriers and associated enablers identified in this study are addressed, there are likely to be positive implications not only for antenatal retesting rates, but also for prenatal care overall and the quality of HIV-related care services provided to all patients.

We found that various client-level barriers contribute to late initial presentation and not returning to antenatal clinic, or not attending clinic at all. The contributing factors identified in this study corroborate results found in the literature, implicating age that is significantly younger or older than the average reproductive ages (15–49) [18, 19], lack of formal education or knowledge about importance of attending ANC early in the gestational period [20, 21], delays by clients in recognizing their pregnancy [20], and not considering pregnancy a health condition that should be treated at a health facility [22]. Addressing late presentation and not returning to antenatal clinic may have benefits for other maternal and child health services in addition to PMTCT, including early folic acid and iron supplementation [23], better malaria prevention [24, 25], ability to complete syphilis treatment before delivery [26], and lower risk of delivery complications for HIV-positive women [27].

While addressing client-level barriers may be important, Ferlie and Shortell suggest that strategies focusing on individuals alone are seldom effective in an attempt to improve policy implementation [16]. At the provider and facility levels, we found that heavy and inconsistent client volume put an emotional strain on providers, contributing ultimately to burnout. Our results indicate that an individualized solution to each clinic is required; some clinics may need additional dedicated HIV testing and counseling personnel while others may need to have their clinicians performing HIV testing alongside their regular duties, thus providing integrated care.

Finally, at the health system level, occasional stock-outs of HIV test kits was identified as the most important barrier to consistently offering repeat HIV testing services. Challenges with supply chain issues are common in countries with a developing infrastructure. Studies show that in addition to impacting HIV testing and counseling programs [28], supply chain issues also affect effective integration of HIV and antenatal services [29], entry and engagement into the HIV continuum of care [30], avoidance of antiretroviral therapy interruptions [31], and provider compliance with HIV care guidelines [32]. Correlates of these supply chain issues include lack of a national stock buffering capacity and long delays from facilities submitting orders to receipt of requested supplies [33, 34], although our data suggest that the move to electronic ordering in Kenya has reduced delays. Additionally, to address the challenges associated with identifying when patients are eligible to receive retesting, while electronic medical records may be ideal, a more realistic goal may be to modify existing registers and booklets to allow for easier longitudinal follow-up.

While several of the barriers and enablers identified in this study are relevant to other clinic services provided, such as offering the initial HIV test at ANC, we felt that several factors uniquely impact repeat HIV testing. In order for retesting to take place, clients need to return to ANC. Therefore barriers influencing discontinued ANC visits, such as being disappointed with long clinic wait times or engaging in seasonal/migratory work, directly put clients at risk of slipping through the holes in HIV care net. One enabler that we identified which may combat this barrier could be the positive testimony of prior clients. This concept has been powerfully employed in the form of “mentor mothers,” HIV-positive women who have been through PMTCT services and serve as peer counselors in antenatal care settings [35, 36]. Another barrier that seemed to be more problematic for repeat testing than initial testing was the lack of HIV test kits, since providers preferentially used limited supplies for initial testing.

The current study has several strengths including the range of perspectives that the participants represented, from direct full-time clinical providers to mid-level program technical advisors and upper-level management. While it is not possible to capture the full scope of possible healthcare provider populations using a sample of three healthcare facilities, the providers at these clinics are likely to reflect those working at rural government health facilities in Nyanza province in general.

Despite these strengths, the study has several limitations. Given that our sample did not include clients or health system administrators, caution should be taken when interpreting barriers and enablers presented at these socioecological levels. Our results may also have limited applicability to urban settings, where the patient population may have easier access to antenatal clinics and greater educational attainment or understanding of the value of early and continued prenatal visits. Additionally, urban facilities may have an appointment system to regulate patient visit dates and times and thus provider workload. The health infrastructure in urban areas may also allow for easier to access to backup HIV test kits and be more conducive to implementation of an electronic medical record system. Finally, the government clinics chosen are all supported by Family AIDS Care Education and Services, a program that provides a level of mentoring and support for PMTCT that other neighboring facilities may not have.

While the providers and managers were able to comment on barriers at the provider and facility levels respectively, more research may need to be conducted to better understand the client perspectives on the barriers they experience to repeat testing. Similarly, investigating perspectives from Ministry of Health and other government-level officials may be valuable for further understanding health system implementation challenges.

## Conclusions

While providers and managers of antenatal care clinics expressed the importance of repeat HIV testing in the key population of pregnant women – both for the sake of the mother and the child, they also shared concerns about barriers at the client, provider, facility, and health system levels that prevented them retesting all pregnant women. In order to meet international and national goals of eliminating mother to child transmission of HIV, a multi-faceted intervention that addresses the barriers and capitalizes on the enablers identified may be required improve antenatal retesting rates. Further research into the implementation challenges of such an intervention will be valuable for facility coordinators, health system administrators, and policy makers.
